# Whole-genome analysis of probiotic product isolates reveals the presence of genes related to antimicrobial resistance, virulence factors, and toxic metabolites, posing potential health risks

**DOI:** 10.1186/s12864-021-07539-9

**Published:** 2021-03-24

**Authors:** Ying Wang, Qian Liang, Bian Lu, Hong Shen, Shuyan Liu, Ya Shi, Sebastian Leptihn, Hong Li, Jin Wei, Chengzhi Liu, Hailong Xiao, Xiaoling Zheng, Chao Liu, Huan Chen

**Affiliations:** 1 Key laboratory of Microbial technology and Bioinformatics of Zhejiang Province, Zhejiang Institute of Microbiology, Hangzhou, 310012 Zhejiang China; 2 Xiaoshan Center for Disease Control and Prevention, Hangzhou, 311201 Zhejiang China; 3 NMPA Key laboratory for Testing and Risk Warning of Pharmaceutical Microbiology, Biological Inspection Department, Zhejiang Institute for Food and Drug Control, Hangzhou, 310052 Zhejiang China; 4 Dalian Customs District, Dalian, 116001 Liaoning China; 5 Zhejiang University-University of Edinburgh Institute, School of Medicine, Zhejiang University, Hangzhou, 310058 Zhejiang China; 6 China National Accreditation Institute for Conformity Assessment, Beijing, 100062 China; 7 Nordkapp Medical Group, Hangzhou, 311121 Zhejiang China; 8 Hangzhou Institute for Food and Drug Control, Hangzhou, 310018 Zhejiang China; 9 Department of Orthopaedics, Sir Run Run Shaw Hospital, School of Medicine, Zhejiang University, Hangzhou, 310009 Zhejiang China

**Keywords:** Probiotic, Health risk, Whole genome analysis, Antimicrobial resistance, Instability

## Abstract

**Background:**

Safety issues of probiotic products have been reported frequently in recent years. Ten bacterial strains isolated from seven commercial probiotic products on market were evaluated for their safety, by whole-genome analysis.

**Results:**

We found that the bacterial species of three probiotic products were incorrectly labeled. Furthermore, six probiotic product isolates (PPS) contained genes for the production of toxic metabolites, while another three strains contained virulence genes, which might pose a potential health risk. In addition, three of them have drug-resistance genes, among which two strains potentially displayed multidrug resistance. One isolate has in silico predicted transferable genes responsible for toxic metabolite production, and they could potentially transfer to human gut microflora or environmental bacteria. Isolates of *Lactobacillus rhamnosus* and *Bifidobacterium animalis* subsp. *lactis* are associated with low risk for human consumption. Based on a comparative genome analysis, we found that the isolated *Enterococcus faecium* TK-P5D clustered with a well-defined probiotic strain, while *E. faecalis* TK-P4B clustered with a pathogenic strain.

**Conclusions:**

Our work clearly illustrates that whole-genome analysis is a useful method to evaluate the quality and safety of probiotic products. Regulatory quality control and stringent regulations on probiotic products are needed to ensure safe consumption and protect human health.

**Supplementary Information:**

The online version contains supplementary material available at 10.1186/s12864-021-07539-9.

## Background

The global market for probiotic products is growing rapidly and estimated to reach 3.5 billion US dollars by 2026 (https://www.gminsights.com/pressrelease/probiotics-market). The widely accepted definition of probiotics is “live microorganisms which when administered in adequate amounts confer a health benefit on the host” [1]. Strains of *Lactobacillus*, *Bifidobacterium* and *Streptococcus* are commonly used as probiotics in foods or feed additives, and strains of *Enterococcus* in “Live biotherapeutic products (LBP)” or “Microecologics for therapeutic use” [2]. According to the “Guidelines for the Evaluation of Probiotics in Food” [1], multiple assessments are essential to demonstrate the safety and health benefits of probiotic strains, which include the assessment of antibiotic resistance, toxin production, hemolytic activity, metabolic activity, and adverse effects.

Though the safety evaluation of probiotic products outlined in the guideline has been accepted as a recommendation and cited frequently, such examination has not been defined as legal requirement in the world. However, safety issues of probiotic products have been reported frequently in recent years. Firstly, the inaccurate labeling can be caused by incorrect taxonomic identification of probiotic strains [3] or contamination [4], seems to be attributed to the limitations of traditional microbiological identification and detection methodology. Secondly, previous research showed that probiotic genome variation would affect probiotic functionality, and the quality assurance and control measures targeting genome stability in probiotic strains are necessary, especially mobile genetic elements [5]. Thirdly, antimicrobial resistance (AMR) genes and virulence factors (VFs) are harmful to human, which needs to be monitored in the screening of probiotics [6]. As we know, the horizontal transfer of AMR genes would accelerate the AMR crisis [7]. Furthermore, the agricultural probiotic products containing VFs can lead to the pathogenic transfer from farms to humans [8]. Fourthly, common bacterial toxic metabolites that are harmful to human health also should be screened, including hemolysins, D-lactic acid (D-lactate), biogenic amines, involving key enzymes such as nitroreductase, amino acid decarboxylase enzyme, and azoreductases [7, 9, 10].

Taken together, the whole-genome analysis is an expected method for accurate identification and safety evaluation [11], which could resolve the rising concerns about the risks of probiotic products on human health [4, 10, 12]. In 2019, China’s State Administration for Market Regulation (SAMR) published the “Application and Evaluation of Probiotic Health Food” and “Health Food Strain Pathogenicity Evaluation Procedure Standard”. These two drafts of public review and comment declare the importance of whole-genome sequencing analysis of probiotics (http://www.samr.gov.cn/hd/zjjg/201907/t20190715_303461.html).

In this study, ten strains were isolated from seven commercial probiotic products, including *Lactobacillus*, *Bifidobacterium*, *Streptococcus*, and *Enterococcus*. We performed strain-level identification, assessed the presence of transferable AMR genes and VF genes, and evaluated the genomic instability of the isolates. Furthermore, we have carried out comparative genome analysis to two *Enterococcus* strains, and results showed that *E. faecalis* isolate was related with the pathogenic strain, while *E. faecium* isolate clustered with probiotic strain.

## Results

### Isolation and identification

Of the seven probiotic products (product number: P2, P3, P4, P5, J6, J7, and F8), bacterial species information was included in their product specification (Table S1), while none of them had bacterial strains information.

#### Whole genome sequencing

In order to characterize the probiotic product isolates (PPS) and their potential risks on consumers, we isolated single bacterial colonies by conventional plate streaking. According to the cell morphology and the colonies 16S rDNA sequence, ten different candidate isolates were selected for whole genome sequencing. The summary data of whole-genome sequencing on the Nanopore GridION platform and the HiSeq Xten platform were shown in Table S2 and Table S3, respectively. Except for that of the isolate TK-P3A, all genome assemblies were complete genomes, and they are publicly available in the NCBI database (Table S4, PRJNA579198).

#### Genome-based identification and mislabeling

According to the ANI calculation, ten bacterial isolates were assigned to *L. rhamnosus*, *B. animalis* subsp. *lactis*, *L. helveticus*, *L. plantarum*, *S. thermophilus*, *E. faecalis*, *E. faecium*, *L. delbrueckii*, *L. reuteri*, and *L. paracasei*, respectively (Table 1). Compared with the reference strain *E. faecium* NCTC 7171, the ANI value of TK-P5D was less than 95 (94.86) (Table S5), whereas both rMLST and TYGS identified TK-P5D as *E. faecium* at the species level. Species identification by rMLST was consistent with that by the ANI value, and it was also consistent with that by TYGS except for TK-P3A. Among the ten isolates, three were inconsistent with their corresponding product labels, based on whole genome identification.

**Table 1:** Identification of bacterial isolates

Isolates	Probiotics Product	Species Identification			Consistency with product label	Probiotics declared on the product label	Strain typing
		ANI^a^	rMLST^**b**^	TYGS^**c**^			
TK-F8B	F8	*L. rhamnosus*	*L. rhamnosus*	*L. rhamnosus*	Consistency	*L. rhamnosus* *B. lactobacillus* *L. reuteri* *B. animalis*	none
TK-J6A	J6	*B. animalis* subsp. *lactis*	*B. animalis*	*B. animalis*	Inconsistency	*B. longum* *B. bifidum* *S. thermophilus* *L. acidophilus* *L. delbrueckii subspecies bulgaricus*	*B. animalis* subsp. *lactis* B420
TK-J7A	J7	*L. helveticus*	*L. helveticus*	*L. helveticus*	Consistency	*L. helveticus* *B. bifidum* *B. infantis*	none
TK-P2A	P2	*L. plantarum*	*L. plantarum*	*L. plantarum*	Inconsistency	*B. longum* *L. acidophilus* *E. faecalis*	none
TK-P3A	P3	*S. thermophilus*	*S. thermophilus*	*S. salivarius* [Unreliable identification]	Consistency	*B. longum* *L. delbrueckii subsp. bulgaricu* NQ-2508 *S. thermophilus*	none
TK-P4B	P4	*E. faecalis*	*E. faecalis*	*E. faecalis*	Consistency	*B. infantis* *L. acidophilus* *E. faecalis* *B. cereus*	*E. faecalis* ST745
TK-P5D	P5	*E. faecium*	*E. faecium*	*E. faecium* [potential new species]	Consistency	*B. subtilis* *E. faecium*	*E. faecium* ST812
P3MRA	P3	*L. delbrueckii*	*L. delbrueckii*	*L. delbrueckii*	Consistency	*B. longum* *L. delbrueckii subsp. bulgaricus* NQ-2508 *S. thermophilus*	none
TK-F8A	F8	*L. reuteri*	*L. reuteri*	*L. reuteri*	Consistency	*L. rhamnosus* *B. lactobacillus* *L. reuteri* *B. animalis*	none
TK-P4A	P4	*L. paracasei*	*L. paracasei*	*L. paracasei*	Inconsistency	*B. infantis* *L. acidophilus* *E. faecalis* *B. cereus*	none

^a^Species identification based on whole genome average nucleotide identity (ANI); ^b^ Species identification based whole genome using rMLST; ^c^ Species identification based whole genome using TYGS

#### Identification at strain-level

Two methods were applied to strain typing. Since the SNP distances has been used to measure genetic relatedness among isolates and strain typing [13–15]. Firstly, we calculated the SNP distances between each PPS genome and all published genomes of the same species, which were downloaded from the NCBI database (Table S6). Results showed that only TK-J6A has the minimum SNP distances (17 bp) with a probiotic strain *B. animalis* subsp. lactis B420, which are similar and can meet the threshold (< 21 bp) suggested by the Food and Drug Administration (FDA) [15, 16]. For other PPSs, the minimum SNP distances are all more than or equal to 40 bp (40-88 bp), not enough for strain typing. In addition, the web-based PubMLST.org was used for strain typing, only three PPS (TK-P3A, TK-P5D, TK-P4B) can be assigned to strain type (two known and one new) (Table S7).

### Safety assessment

#### Antimicrobial resistance and virulence factors

**Fig. 1:**
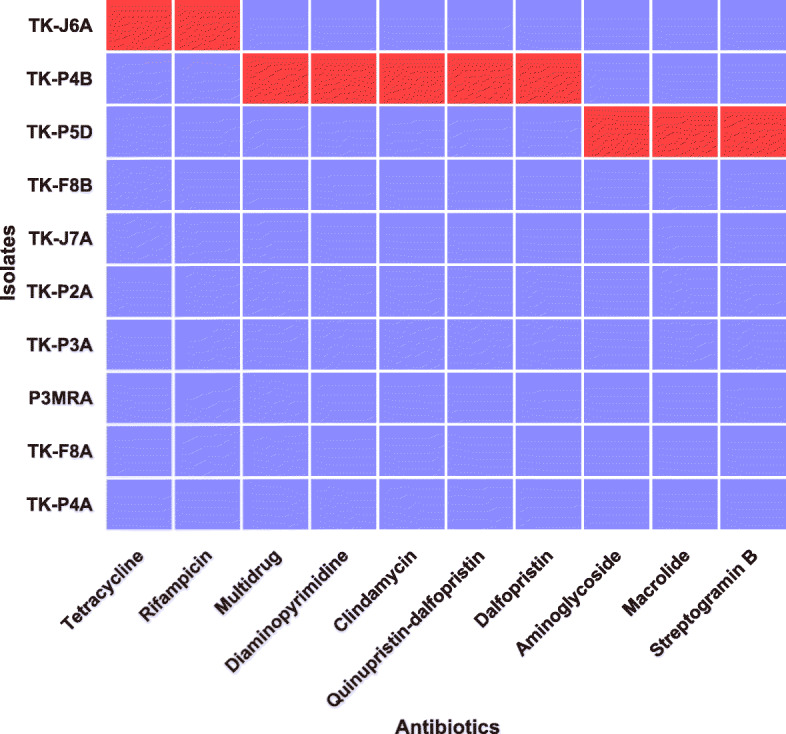
Heatmap showing the AMR genes in all isolates. Red color indicates the presence of intrinsic AMR genes, while blue color indicates their absence

**Fig. 2:**
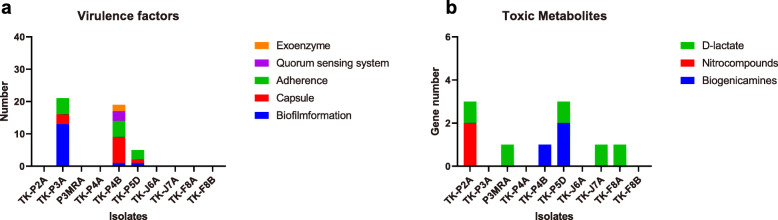
Virulence factors and toxic metabolites detected in all isolates (**a**) virulence factors; (**b**) toxic metabolites

AMR genes were just identified in *B. animalis* subsp. *lactis* TK-J6A, *E. faecalis* TK-P4B, and *E. faecium* TK-P5D (Fig. 1, Table 2, Table S8). For VFs, 21, 19, and 8 virulence genes were identified in the genomes of *S. thermophilus* TK-P3A, *E. faecalis* TK-P4B, and *E. faecium* TK-P5D, respectively (Fig. 2a, Table S9). In the genome of *S. thermophilus* TK-P3A, we identified ssp-5 agglutinin receptor genes, which are involved in polysaccharide and exopolysaccharide biosynthesis, a sortase gene, as well as genes encoding choline- and fibronectin-binding proteins and streptococcal plasmin receptor/GAPDH (Table S9). In *E. faecalis* TK-P4B, we detected *fsr* loci (*fsrA*, *fsrB*, and *fsrC*), a virulence gene cluster associated with capsule synthesis, Ebp pili expression, fibrinogen binding protein synthesis, as well as the expression of gelatinase, hyaluronidase, and SprE. In the genome of *E. faecium* TK-P5D, we identified a virulence gene encoding phosphatidate cytidylyltransferase, a well-recognized virulence factor in enterococci. Whereas, no genes encoding recognized virulence factors were identified in *Bifidobacterium* and *Lactobacillus* isolates.

**Table 2:** The summary of safety risks in all isolates

Isolates	Toxic Metabolites	Virulence factors	Antibiotic resistance	Number of active prophages	Number of transposons	Number of plasmids
*L. rhamnosus* TK-F8B	none	none	none	0	0	2
*B. animalis* subsp. lactis TK-J6A	none	none	tetracycline, rifamycin	0	0	0
*L. helveticus* TK-J7A	D-lactate	none	none	0	0	2
*L. plantarum* TK-P2A	D-lactate	none	none	0	0	2
*S. thermophilus* TK-P3A	none	adhesion, biofilm formation, virulence	none	0	0	1
*E. faecalis* TK-P4B	biogenic amines	biofilm formation, virulence	multidrug resistance	1	1	3
*E. faecium* TK-P5D	biogenic amines	adhesion, biofilm formation, virulence	multidrug resistance	3	0	1
*L. delbrueckii* P3MRA	D-lactate, nitrocompounds	none	none	0	0	0
*L. reuteri* TK-F8A	D-lactate	none	none	5	0	0
*L. paracasei* TK-P4A	none	none	none	4	0	3

#### Toxic metabolites

We next performed a BLAST search against protein sequences of the isolates to identify whether they can produce metabolites that are toxic for human health. No genes associated with toxic metabolite production were found in *L. rhamnosus* TK-F8B, *B. animalis* subsp. *lactis* TK-J6A, *S. thermophilus* TK-P3A, and *L. paracasei* TK-P4A (Fig. 2b, Table S10). However, both *Enterococcus* isolates contained key genes that are associated with biogenic amine synthesis. In addition, four *Lactobacillus* isolates (*L. plantarum* TK-P2A, *L. delbrueckii* P3MRA, *L. helveticus* TK-J7A and *L. reuteri* TK-F8A) contained key genes for D-lactate synthesis, and one *Lactobacillus* isolate (*L. plantarum* TK-P2A) contained key genes associated with nitro compounds production.

### Genome instability

#### ISs and transposons

**Fig. 3:**
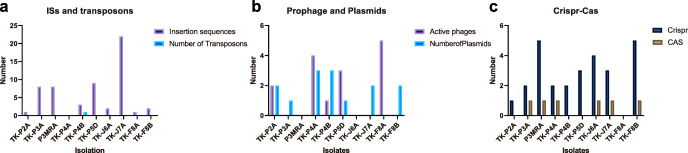
Mobile elements and associated genetic elements detected in all isolates (**a**) Insertion sequences and transposons; (**b**) prophages and plasmids; (c) Crispr-Cas systems

**Fig. 4:**
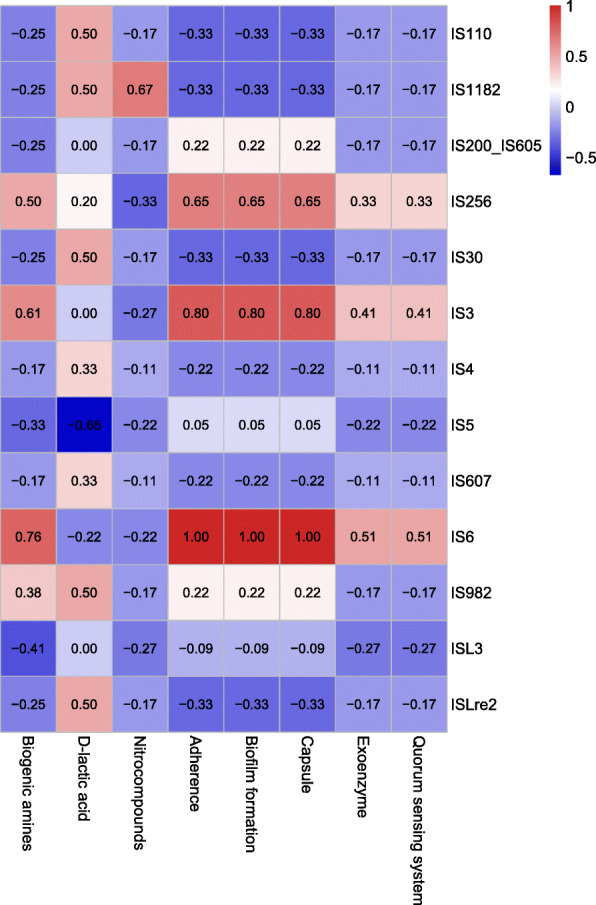
Heatmap showing correlation between IS elements and virulence factors (including toxic metabolite genes) found across the genomes of all isolates. Red color indicates a strong positive correlation while blue color indicates a strong negative correlation

ISs are genetic mobile elements that allow embedded genes to spread among microbes through horizontal gene transfer. The IS elements from 14 families (E < 1e-5, coverage > 60%, and identity > 90%) were found in the genomes of nine isolates (Fig. 3a, Table S11). Moreover, our results indicated that specific IS elements (IS6 and IS3) had strong correlations with the virulence factors for capsule or biofilm formation and bacterial adherence (Fig. 4), suggesting that IS6 and IS3 contribute in the transfer of these virulence factors. In the genome of *L. delbrueckii* P3MRA, we also identified that D-lactate dehydrogenase gene (GFB67_00380) was located at the downstream of IS7, indicating this toxic metabolite gene might be regulated by IS7. In the genome of *L. helveticus* TK-J7A, a hypothetical gene (GFB61_02125) was located between ISLhe7 and ISLjo1, and a glycosyltransferase gene (GFB67_08805) was located between ISLdl2 and ISL5, indicating potential genetic instability.

Similar to ISs, transposons usually embed more than one accessory genes for antibiotic resistance or microbial virulence. According to the results from ICEberg 2.0 (http://db-mml.sjtu.edu.cn/ICEberg/), only a multidrug resistance transposon was identified in the genome of *E. faecalis* TK-P4B (Fig. 3a, Table S12), and no AMR has been found to be associated with this transposon.

#### Prophages and plasmids

Several lysogenic prophages contain genes that contribute to microbial motility and biofilm formation. Therefore, we analyzed the genomes of the isolates to search for genome-embedded phage genes using the program Prophage Hunter (https://pro-hunter.bgi.com/). We found that the genomes of *L. paracasei* TK-P4A, *L. reuteri* TK-F8A, *E. faecium* TK-P5D, and *E. faecalis* TK-P4B contained active prophage genes (Fig. 3b, Table S13). Among the four active prophage genes detected in *L. paracasei* TK-P4A, one was the most closely related to an *Enterococcus* phage. Among the five active prophage genes detected in *L. reuteri* TK-F8A, two were closely related to *Staphylococcus* phages. One active prophage gene related to a *Staphylococcus* phage was detected in *E. faecalis* TK-P4B, and one related to a *Streptococcus* phage was detected in *E. faecium* TK-P5D. No VFs or AMRs has been found in the active prophages.

Plasmids play a major role in frequent genetic information (e.g. AMR) exchanges in prokaryotes. Therefore, we also characterized the plasmids in the ten PPS. Based on hybridized assemblies, we found that seven isolates contained such epigenetic elements. The plasmid in *E. faecium* TK-P5D encodes four virulence factors that are responsible for biofilm formation, which could potentially result in virulence factor transfer towards gut microflora or environmental microbes. No toxic metabolite-associated genes or AMR genes were found in the plasmids of these isolates (Fig. 3b, Table S14).

#### Crispr/Cas systems and genomic islands

Crispr/Cas systems in bacteria contribute to viral defense, and the likelihood of gene (e.g. AMR genes) acquisition from bacteriophages might increase if the bacterial strain contains no such system. Crispr/Cas systems were identified in six isolates, including *L. rhamnosus* TK-F8B, *B. animalis* subsp. *lactis* TK-J6A, *L. helveticus* TK-J7A, *S. thermophilus* TK-P3A, *L. delbrueckii* P3MRA, and *L. paracasei* TK-P4A (Fig. 3c, Table S15). Whereas, *E. faecalis* TK-P4B, *E. faecium* TK-P5D, and *L. plantarum* TK-P2A contained orphan Crispr arrays without Cas genes.

Genomic islands that contain large quantities of associated genes in clusters were also searched, but they were not identified in any of the bacterial isolates.

#### Analysis of transferable AMRs and VFs

We further assessed the AMR and VF genes for potential horizontal transfer towards other bacteria, and analyzed the correlations between these genes and mobile elements, based on genomic position and correlation analysis. No AMR genes have been found to be located in mobile genetic elements, and no strong correlation was found between AMR genes and ISs, indicating that all AMRs are intrinsic. For VFs, results showed that VFs associated with biofilm formation and adherence in TK-P3A, TK-P4B and TK-P5D, showed high correlation with mobile elements, indicating IS3 and IS4 might play important roles in transfer of these VFs (Fig. 4).

### Comparison of probiotic, non-pathogenic and pathogenic strains

**Fig. 5:**
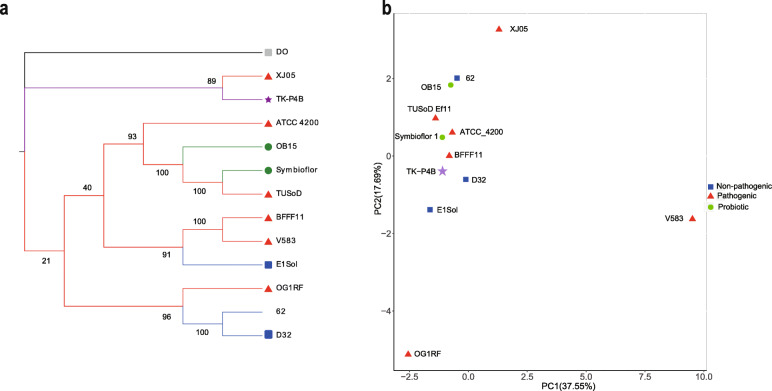
Phylogenetic analysis of the genomes of the well-defined pathogenic, non-pathogenic, and the probiotic *E. faecalis* strains with the TK-P4B: **a** Phylogenetic tree of *E. faecalis* genome sequences based on analysis of core genes, and classification of strains are grouped into probiotic (green circle), non-pathogenic (red triangle), probiotic isolate evaluated in this study (purple star), and the outgroup (gray circle) groups; **b** PCA analysis of *E. faecalis* genome sequences based on presence or absence of mobile elements, and genes responsible for virulence factors, toxic metabolites and antibiotic resistance, and classification of strains are grouped into probiotic (green circle), non-pathogenic (red triangle), probiotic isolate evaluated in this study (purple star)

Since core genes-based phylogenetic reconstruction can be applied to find potential probiotic candidates [17], the genomes of *E. faecalis* TK-P4B and *E. faecium* TK-P5D were further compared to well-defined *E. faecalis* and *E. faecium* strains, respectively. For *E. faecalis* TK-P4B, two *E. faecalis* probiotic strains (Symbioflor 1 Clone DSM 16431 and OB15), two non-pathogenic *E. faecalis* strains (62 and E1Sol), six pathogenic *E. faecalis* strains (BFFF11, XJ05, OG1RF, TUSoD Ef11, ATCC 4200, and V583), and *E. faecium* strain DO (as an outgroup) were compared. A phylogenetic tree was generated using the core genes by the Maximum Likelihood method (Fig. 5a). We found that TK-P4B was clustered with the pathogenic strain XJ05. Furthermore, PCA was performed based on the key genes associated with virulence factors and the presence of mobile elements, which confirmed the clustering with pathogenic strain (Fig. 5b). The assessment based on Euclidean distances revealed that TK-P4B was more closely related with the pathogenic strain BFFF11 while less related with the non-pathogenic strain E1Sol.

**Fig. 6:**
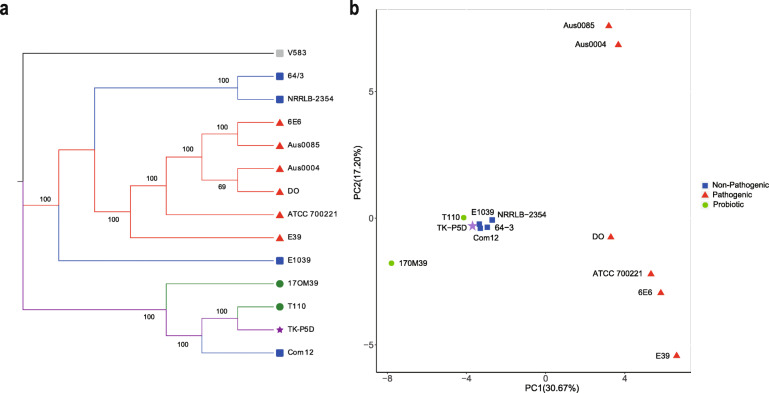
Phylogenetic analysis of the genomes of well-defined pathogenic, non-pathogenic and the *E. faecium* strains with TK-P5D: **a** Phylogenetic tree of *E. faecium* genome sequences based on analysis of core genes, and classification of strains are grouped into probiotic (green circle), non-pathogenic (red triangle), probiotic isolate evaluated in this study (purple star), and the outgroup (gray circle) groups; **b** PCA analysis of *E. faecium* genome sequences based on presence or absence of mobile elements, and genes responsible for virulence factors, toxic metabolites and antibiotic resistance

In addition, two well-defined *E. faecium* probiotic strains (17OM39 and T110), four non-pathogenic *E. faecium* strains (64/3, NRRLB-2354, E1039, and Com 12), six pathogenic *E. faecium* strains (6E6, Aus0085, Aus0004, DO, ATCC 700221, and E39), and *E. faecalis* V583 (as an outgroup) were compared with the *E. faecium* TK-P5D by phylogenetic analysis (Fig. 6a). The fourteen strains were clustered into three distinct clusters with high bootstrap support (bootstrap = 100). We found that TK-P5D was clustered with 17OM39, T110, and one non-pathogenic strain Com 12. These results were further confirmed by PCA based on the key genes associated with virulence factors and the presence of mobile elements (Fig. 6b).

## Discussion

The manufacturers are required to accurately indicate the bacterial strains on commercial probiotic products for human consumption, to comply with the government regulations and guidelines [1]. However, none of the seven probiotic products investigated in this study indicated their specific bacterial strain information. In this study, ten isolates genome sequence were obtained, and result revealed that the species-level identification of bacterial isolates from J6, P2, and P4 products were inconsistent with their label descriptions. For accurate species identification, whole genome sequence should be applied.

Whole-genome SNP distance has been accepted as a reliable method for assessing the genetic relatedness among bacterial isolates in recent years [15]. The SNP distance analysis revealed only the mislabeling isolate TK-J6A could be assigned to the probiotic strain *B. animali*s subsp. *lactis* B420, indicating it is a potential probiotic strain. In addition, we used the MLST method for strain typing, however, due to the lack of MLST schemes on PubMLST.org website, only two *Enterococcus* isolates (TK-P4B and TK-P5D) were assigned to known ST types and the *S. thermophilus* TK-P3A was assigned to a new ST type. The major restrictive factor for strain-level identification of PPS employing SNP distances or MLST is the limited number of published genomic sequences. It is indicated that a comprehensive genome database needs to be constructed for safety evaluation, strain typing and property rights protection, especially in probiotic area.

In Qualified presumption of safety (QPS) criteria, probiotic assessment includes identity, body of assessment, safety concern and antimicrobial resistance [18]. Ideally, probiotics should be susceptible to at least two antibiotics, or should not carry intrinsic antimicrobial resistance [19]. Our study revealed that several PPS contained AMR genes that can potentially render the effectiveness of antibiotics. Previous reports have shown that the isolates of *B. animalis* subsp. *lactis* contained *tet* (W) genes for tetracycline resistance, but these genes had low transmissibility [20, 21]. Moreover, the well-defined AMRs detected in *E. faecium* TK-P5D and *E. faecalis* TK-P4B, indicating that both strains are multidrug-resistant [22, 23]. Although AMRs showed non-potential transferability and seems to be intrinsic, the two *Enterococcus* isolates with multidrug-resistance genes may potentially induce safety issues, when they obtain external virulence genes with a very small probability.

VFs related to capsule or biofilm formation and bacterial adherence were detected in the genome of *S. thermophilus* TK-P3A, which also be reported to facilitate probiotic survival in gastrointestinal tract by strengthening acid resistance and intestinal colonization [17, 24–30]. More research needs to clarify the underlying mechanisms of adaption and pathogenicity. On the other hand, the well-characterized VFs (e.g., *fsr* loci, gene cluster encoding pili) identified in *E. faecalis* TK-P4B and *E. faecium* TK-P5D, indicating their potential safety risk for human consumption [31–34]. The strong correlation of ISs and VFs in TK-3A, TK-P4B and TK-P5D indicates the possibility of acquisition and loss of VFs. The significant positive correlation between ISs and pathogenic genes (VF and toxic metabolites associated) suggests that IS6 and IS3 may play important roles in VF acquisition and loss. Further studies need to be carried out to characterize gene transfer events from oral probiotics to enterobacteria. We found that four *Lactobacillus* isolates can produce D-lactic acid, while two *Enterococcus* isolates can produce nitro compounds. It is possible that they only produce these toxic metabolites under certain conditions e.g. in specific hosts or under particular stimulation, which is difficult to be characterized by conventional phenotypical studies [35].

The absence of plasmids, active prophage genes, or transposons in *B. animalis* subsp. *lactis* TK-J6A indicates its genome is stable. The absence of CRISPR/Cas systems might potentially increase the likelihood of AMR acquisition from bacteriophages or plasmids [36]. *E. faecalis* TK-P4B, *E. faecium* TK-P5D, and *L. reuteri* TK-F8A contained no CRISPR/Cas system, that’s maybe the reason for the presence of embedded active prophage gene sequences, multidrug resistance-associated transposons, and/or virulence-associated plasmids in these PPS.

No VF, transferable AMR or few mobile genetic elements were found in the *L. rhamnosus* TK-F8B and *B. animalis* subsp. *lactis* TK-J6A isolates, suggesting the two strains are good probiotic candidates. On the other hand, the two *Enterococcus* isolates (commonly used in LBPs or microecologics for therapeutic use), potentially display drug resistance, virulence factors, toxic metabolites, and genetic instability. Furthermore, considering that the TK-P5D isolate clustered with a well-defined *E. faecium* probiotic strain, and the TK-P4B isolate clustered with a pathogenic strain, suggesting TK-P5D is a potential probiotic, while TK-P4B is potential a pathogenic strain, if clustering based on pathogenic, NPNP or probiotic groups as previously reported [17]. We will further discuss the safety of the strain with the P4 product manufacturer, combined with more phenotype data, to further explore the safety risks of the strain TK-P4B.

Although there is a certain gap between phenotype and genotype, we all know that phenotype is the expression of genotype under specific conditions. Therefore, the safety evaluation of PPS based on whole genome analysis will contribute to the healthy development of probiotic industry.

## Conclusion

In this study, we uncover several risk factors in the commercial probiotic products by whole genome analysis, suggesting the probiotic products on market require stringent regulations with systematic testing and quality control. For safety evaluation of probiotic products, whole-genome analysis is a promising technique, and its performance will be further improved as the genomic and phenotypic data are growing rapidly.

## Methods

### Probiotic products and bacterial isolation

Seven commercial probiotic products from different countries licensed for human consumption, including two health supplements, three Over-the-Counter drugs, one prescription drug, and one solid beverage, were purchased from online stores, online pharmacies, or local drugstores in China (Hangzhou, Zhejiang Province, China) in 2019, and immediately stored at 4 °C. The forms of these probiotic products included powders, granules, and tablets.

The powders and granules were directly dissolved in 20 mL PBS for mixing, while the tablets were milled into powders and then dissolved in 20 mL PBS for mixing. An aliquot of 100 μL diluted mixture was applied to agar plates and cultured at 36 °C for 48 h to 72 h under anaerobic condition. Isolates of *Enterococcus* were cultured on Tryptic Soy Agar (TSA) (Hopebio, HB0177) under aerobic conditions. Isolates of *Lactobacillus*, *Bifidobacterium*, and *Streptococcus* were cultured on de Man, Rogosa & Sharpe (MRS) or *Lactobacillus* Selective (LBS) agar (Hopebio, HB0385), MRS agar (Hopebio, HB0384), and Modified Chalmers (MC) agar (Hopebio, HB0383), respectively, under anaerobic condition. According to the cell morphology and 16S rDNA sequence, the different candidate isolates were ready for whole genome sequencing.

### Whole-genome sequencing

The candidate isolates were passaged on their respective fresh agar plates for sub-culturing at 36 °C for 48 to 72 h. The isolated strains were subsequently incubated with 100 μL Lysozyme (10 mg/mL) (Sigma, 62,970) for 1 h at 37 °C, and then the total DNA was extracted using Puregene Yeast/Bact Kit (Qiagen, 1,042,607) according to the reference manual. The quality and quantity of bacterial genomic DNA were evaluated by electrophoresis on a 1% agarose gel, a NanoDrop2000 (Thermo Scientific, Waltham, USA), and a Qubit 4 Fluorometer (Thermo Scientific, Waltham, USA).

Whole-genome sequencing was performed on both the Illumina HiSeq Xten platform (Illumina, California, USA) at GeneSeeq Co., Ltd. (Nanjing, China) and the Oxford Nanopore Technology’s (ONT) GridION sequencing platform (ONT, Oxford, United Kingdom) at Zhejiang Tianke Technology Co., Ltd. (Hangzhou, China). For Illumina sequencing, 350 bp DNA libraries were prepared using TruPrep DNA Library Prep kit V2 (Illumina, San Diego, USA) according to the manufacturer’s protocol. For SMART sequencing, 20–40 kb DNA libraries were constructed using SQK-LSK109 Ligation Sequencing kit (ONT, Oxford, United Kingdom) according to the manufacturer’s protocol. Raw data from the Illumina sequencing were cleaned by removing the reads with low quality or adapter contamination. Raw data from the SMART sequencing were cleaned by removing the reads with mean_qscore_template <7 or length <1000 bp, and then corrected and trimmed using the Canu package (version 1.7.11) with default parameters. Subsequent genomic assembly was performed with all sequencing data using Unicycler (v0.4.5) software [37] (−-min_fasta_length 500, −-no_correct, −-kmers 57,65,69,73,79,85, −t 16, −-mode bold, −-depth_filter 0.28, −-keep 3). The sequences of the complete genome and plasmids of the bacterial isolates have been deposited in NCBI. Bacterial genomic information was downloaded from NCBI. The average nucleotide identity (ANI) was calculated using fastANI [38].

The final assembled genomes including chromosomes and plasmids were annotated using the Prokaryotic Genome Annotation Pipeline (PGAP) algorithm (NCBI, Bethesda, MD, USA).

### Identification

ANI was used for genome-wide comparisons for bacteria identification. The ANI of each assembly against the genome of the reference strain was calculated using FastANI. An ANI > 95% represents the same bacterial species. The Type (Strain) Genome Server (TYGS, https://tygs.dsmz.de) [39] and Ribosomal Multilocus Sequence Typing (rMLST, https://pubmlst.org/bigsdb?db=pubmlst_rmlst_seqdef_kiosk) [40] with whole genome sequences input were also used for species identification of the isolates.

To further identify the taxonomic status of the bacterial isolates at strain level, the single nucleotide polymorphism (SNP) distance was quantified as a measure of strain-relatedness using kSNP3.1.2 [41]. The web-based platform PubMLST.org was used for strain typing-based MLST [42].

### Genome instability

The clustered regularly interspaced short palindromic regions (CRISPR) were identified using the CRISPRCasFinder tools [43]. Putative prophage sequences in the isolates were identified using ProphageHunter (https://pro-hunter.bgi.com/) [44]. Bacterial insertion sequences (ISs) were identified using ISfinder [45]. Horizontal gene transfer was detected using the genomic island tool Islandviewer 4 [46]. Plasmids were verified using Blastn, aligning assemblies to plasmids sequences of NCBI RefSeq database (−evalue 1e-5, Identity> = 95%, query coverage> = 80%).

### Identification of genes related to antibiotic resistance, virulence factors, and toxic metabolites

AMR genes were identified by BLASTp with the Comprehensive Antibiotic Resistance Database (CARD) (http://arpcard.mcmaster.ca/) [47]. Putative virulence genes were identified by BLAST against the Virulence Factors Database (VFDB) (http://www.mgc.ac.cn/VFs/main.htm) [48]. Sequences of genes encoding toxic metabolites (D-lactic acid, nitrocompounds, and biogenic amines)-associated proteins, including D-lactate dehydrogenases, nitro reductases, nitrate reductase, and amino acid decarboxylases, were downloaded from Uniprot (https://www.uniprot.org/) [49]. The presence of toxic metabolites-related genes was searched against the genome of all the isolates by BLASTx. Only the BLAST results showing a cut-off E value of 1e-5, an identity > 80%, and a coverage > 90% were considered.

### Comparative analysis

The whole genome sequences of twelve well-defined E. faecium strains [17, 50] and eleven E. faecalis strains [50–55] were downloaded from NCBI genomes. A total of fourteen genomes of E. faecium (E. faecalis V583 as the outgroup) and thirteen whole genome sequences of E. faecalis (E. faecium DO as the outgroup) were used for phylogenetic analysis, respectively. The core genes in E. faecium or E. faecalis genomes were obtained using BLAST-2.2.22 and OrthoMCL V1.4 [56]. Multiple sequence alignments of the core genes were conducted using MUSCLE v3.8.31 [57], and the phylogenetic analyses were conducted using IQ-TREE (version 1.6.6) [58]. Genes associated with antibiotic resistance, virulence factors, and toxic metabolites were selected for Principal Component Analysis (PCA) by R statistical platform version 3.4.3, and the corresponding figures were generated using ggplot packages. The correlation between IS elements and virulence factors or toxic metabolites was calculated by R statistical platform version 3.4.3, and the corresponding figures were generated using pheatmap package.

## Supplementary Information

**Additional file 1: Table S1.** Probiotic Product Information. **Table S2.** Quality control of sequencing data from smart sequencing. **Table S3.** Quality control of sequencing data from Hiseq Xten platform. **Table S4.** Assemblies of all probiotic isolates. **Table S5.** The maximum ANI between probiotic isolates and type strains. **Table S6.** Strain typing by SNP distance (< 100 bp). **Table S7.** Strain typing by MLST based on whole genome. **Table S8.** Antibiotic resistance of isolates from probiotic products. **Table S9.** Annotated virulence factors in genomes of probiotic isolates. **Table S10.** Annotated toxic metabolites in probiotic isolates. **Table S11.** Insertion sequences in the genomes of all isolates. **Table S12.** Transposon in the genomes of isolates from probiotic products. **Table S13.** Transposon in the genomes of isolates from probiotic products. **Table S14.** Plasmids in the probiotic isolates. **Table S15.** Crispr-Cas systems in the genomes of isolates from probiotic products.

## Data Availability

Additional data can be found in supplementary files. The genome data were submitted to the Genbank database under accession number GCA_015377505.1, GCA_015377745.1, GCA_015377765.1, GCA_015377585.1, GCA_015377485.1, GCA_015377525.1, GCA_015377785.1, GCA_015709185.1, GCA_015377805.1, GCA_015190465.1.

## Abbreviations

*L.*: *Lactobacillus*
*B.*: *Bifidobacterium*
*S.*: *Streptococcus*
*E.*: *Enterococcus*
PPS: Probiotic product isolate
PCA: Principal Component Analysis
