# Supplementary material for: Whole-genome analysis of probiotic product isolates reveals the presence of genes related to antimicrobial resistance, virulence factors, and toxic metabolites, posing potential health risks
Source: BMC Genomics. 2021 Mar 24;22:210. doi: 10.1186/s12864-021-07539-9 (PMC7988973; doi:10.1186/s12864-021-07539-9)
Supplement: Supplementary file 1 — Additional file 1: Table S1. Probiotic Product Information. Table S2. Quality control of sequencing data from smart sequencing. Table S3. Quality control of sequencing data from Hiseq Xten platform. Table S4. Assemblies of all probiotic isolates. Table S5. The maximum ANI between probiotic isolates and type strains. Table S6. Strain typing by SNP distance (< 100 bp). Table S7. Strain typing by MLST based on whole genome. Table S8. Antibiotic resistance of isolates from probiotic products. Table S9. Annotated virulence factors in genomes of probiotic isolates. Table S10. Annotated toxic metabolites in probiotic isolates. Table S11. Insertion sequences in the genomes of all isolates. Table S12. Transposon in the genomes of isolates from probiotic products. Table S13. Transposon in the genomes of isolates from probiotic products. Table S14. Plasmids in the probiotic isolates. Table S15. Crispr-Cas systems in the genomes of isolates from probiotic products. [file 12864_2021_7539_MOESM1_ESM.docx]

# BMC Genomics

**Whole-genome analysis of probiotic product isolates reveals the presence of genes related to antimicrobial resistance, virulence factors, and toxic metabolites, posing potential health risks**

Ying Wang ^1†^, Qian Liang^1†^, Bian Lu ^2†^, Hong Shen ^3^, Shuyan Liu^4^, Ya Shi ^1^, Sebastian Leptihn ^5^, Hong Li ^6^, Jin Wei ^7^, Chengzhi Liu ^1^, Hailong Xiao ^8^, Xiaoling Zheng ^3^, Chao Liu ^9*^, and Huan Chen ^1*^

1 Key laboratory of Microbiol technology and Bioinformatics of Zhejiang Province, Zhejiang Institute of Microbiology, Hangzhou 310012, Zhejiang, China

2 Xiaoshan Center for Disease Control and Prevention, Hangzhou 311201, Zhejiang, China

3 NMPA Key laboratory for Testing and Risk Warning of Pharmaceutical Microbiology, Biological Inspection Department, Zhejiang Institute for Food and Drug Control, Hangzhou 310052, Zhejiang, China

4 Dalian Customs District, Dalian 116001, Liaoning, China

5 Zhejiang University-University of Edinburgh Institute, School of Medicine, Zhejiang University, Hangzhou 310058, Zhejiang, China

6 China National Accreditation Institute for Conformity Assessment, Beijing 100062, China

7 Nordkapp Medical Group, Hangzhou 311121, Zhejiang, China

8 Hangzhou Institute for Food and Drug Control, Hangzhou 310018, Zhejiang, China

9 Department of Orthopaedics, Sir Run Run Shaw Hospital, School of Medicine, Zhejiang University, Hangzhou 310009, Zhejiang, China

***** Correspondence: chenhuan7809@gmail.com; Tel.: +86-571-56021327; liuchaozju@zju.edu.cn; Tel.: + 86 571 87887950

**Table S1.** Probiotic Product Information

| **Number** | **Product form** | **Probiotics declared on labels** | **Classification** | **Production batch number** | **Product name** |
| --- | --- | --- | --- | --- | --- |
| F8 | Powder | *L. rhamnosus*, *B. lactobacillus, L. reuteri*, *B. animalis* | Food | 20180901 | Probiotic bacterium powder |
| J6 | Granules | *B. longum*, *B. bifidum*, *S. thermophilus*, *L. acidophilus*, *L. delbrueckii* subspecies bulgaricus | Health food | 20180611 | Probiotic Granules |
| J7 | Probiotics sachet | *L. helveticus*, *B. bifidum*, *B. infantis* | Food | 810012099 | Probiotics sachet for children |
| P2 | Powder | *B. longum*, *L. acidophilus*, *S. faecalis* | Over-the-Counter drug | 04320180856-2 | Live combined B.,L. and E. Powder，oral |
| P3 | Tablets | *B. longum*, *L. bulgaricus*, *S. thermophilus* | Over-the-Counter drug | 04320180856-2 | Live combined B. and L. Tablets |
| P4 | Tablets | *B. infantis*, *L. acidophilus*, *E. faecalis*, *B. cereus* | Over-the-Counter drug | 201809382 | Combined B., L., E. and B. cereus Tablets, Live |
| P5 | Granules | *B. subtilis*, *E. faecium* | Over-the-Counter drug | 18080014 | Combined B.subtilis and E.Faecium Granules with multivitamines,Live |

**Table S2.**Quality control of sequencing data from smart sequencing

| **Isolates** | **Raw Reads** | **Raw Bases** | **Clean Reads** | | **Clean Bases** | | **Effective Rate(%)** | **Q>20(%)** | **Q>30(%)** |
| --- | --- | --- | --- | --- | --- | --- | --- | --- | --- |
| TK-F8A | 4,171,450 | 625,717,500 | | 3,835,600 | | 560,600,052 | 91.95 | 98.16 | 94.25 |
| TK-F8B | 5,134,488 | 770,173,200 | | 4,749,390 | | 692,852,111 | 92.5 | 98.23 | 94.31 |
| TK-J6A | 19,643,110 | 2,946,466,500 | | 17,571,224 | | 2,509,278,854 | 89.45 | 98.04 | 93.5 |
| TK-J7A | 5,843,716 | 876,557,400 | | 5,385,408 | | 779,706,074 | 92.16 | 98.29 | 94.65 |
| TK-P2A | 4,590,522 | 688,578,300 | | 4,207,100 | | 614,274,505 | 91.65 | 98.07 | 93.92 |
| TK-P3A | 21,656,872 | 3,248,530,800 | | 19,490,238 | | 2,732,665,063 | 90 | 98.5 | 95.14 |
| P3MRA | 11,694,866 | 1,754,229,900 | | 10,671,082 | | 1,545,812,307 | 91.25 | 97.44 | 92.31 |
| TK-P4A | 10,187,802 | 1,528,170,300 | | 9,194,942 | | 1,308,580,143 | 90.25 | 98.18 | 94.12 |
| TK-P4B | 8,525,332 | 1,278,799,800 | | 7,815,156 | | 1,103,031,443 | 91.67 | 98.4 | 94.93 |
| TK-P5D | 11,400,708 | 1,710,106,200 | | 10,782,094 | | 1,572,526,340 | 94.57 | 99.02 | 96.69 |

**Table S3.** Quality control of sequencing data from Hiseq Xten platform

| **Isolates** | **Total raw bases** | **Number of raw reads** | **Mean read length** | **Mean read quality** | **clean bases** | **clean reads** | **Mean clean read Length** | **Mean clean read quality** | **clean read N50 length** | **effective(%)** |
| --- | --- | --- | --- | --- | --- | --- | --- | --- | --- | --- |
| TK-F8A | 697,416,759 | 87,616 | 7,959.90 | 9.9 | 675,018,941 | 71,485 | 9,442.80 | 10 | 15,277 | 96.79 |
| TK-F8B | 829,489,291 | 65,441 | 12,675.40 | 9.8 | 805,055,816 | 56,573 | 14,230.40 | 10 | 23,291 | 97.05 |
| TK-J6A | 629,251,380 | 57,447 | 10,953.60 | 9.6 | 609,720,021 | 48,801 | 12,494.00 | 9.7 | 21,407 | 96.9 |
| TK-J7A | 458,619,809 | 34,188 | 13,414.60 | 10 | 446,839,927 | 29,397 | 15,200.20 | 10.1 | 26,795 | 97.43 |
| TK-P2A | 499,381,720 | 37,776 | 13,219.50 | 9.8 | 483,925,137 | 32,846 | 14,733.20 | 9.9 | 27,483 | 96.9 |
| TK-P3A | 593,173,845 | 67,154 | 8,833.00 | 9.8 | 569,210,040 | 58,079 | 9,800.60 | 9.9 | 14,929 | 95.96 |
| P3MRA | 614,466,469 | 67,837 | 9,058.00 | 9.7 | 595,295,582 | 57,056 | 10,433.50 | 9.8 | 16,476 | 96.88 |
| TK-P4A | 651,531,741 | 44,495 | 14,642.80 | 9.8 | 630,278,689 | 38,753 | 16,264.00 | 9.9 | 26,335 | 96.74 |
| TK-P4B | 459,848,595 | 39,171 | 11,739.50 | 9.9 | 445,517,232 | 34,274 | 12,998.70 | 10 | 20,809 | 96.88 |
| TK-P5D | 587,536,557 | 36,535 | 16,081.50 | 9.9 | 572,012,285 | 32,077 | 17,832.50 | 10 | 29,875 | 97.36 |

**Table S4.** Assemblies of all probiotic isolates

| **Isolates** | **N50(bp)** | **Total_length(bp)** | **Number(>200bp)** | **Number(>2000bp)** | **GC(%)** | **Accession number** |
| --- | --- | --- | --- | --- | --- | --- |
| TK-F8A | 2038911 | 2038911 | 1 | 1 | 38.9 | CP045605 |
| TK-F8B | 3016822 | 3058443 | 3 | 3 | 46.6 | CP045586, CP045587,CP045588 |
| TK-J6A | 1944283 | 1944283 | 1 | 1 | 60.5 | CP045589 |
| TK-J7A | 2133360 | 2163684 | 3 | 3 | 36.8 | CP045590, CP045591, CP045592 |
| TK-P2A | 3199525 | 3210841 | 3 | 3 | 44.6 | CP045593,CP045594, CP045595 |
| TK-P3A | 1989753 | 1993251 | 2 | 2 | 39.1 | CP045596,CP045597 |
| P3MRA | 1871250 | 1871250 | 1 | 1 | 49.7 | CP045604 |
| TK-P4A | 3020185 | 3080275 | 4 | 4 | 46.4 | CP045567,CP045568, CP045569,CP045570 |
| TK-P4B | 2730170 | 2745628 | 4 | 4 | 37.6 | CP045598, CP045599, CP045600, CP045601 |
| TK-P5D | 2724200 | 2802870 | 2 | 2 | 38.4 | CP045602, CP045603 |

**Table S5. The maximum ANI between probiotic isolates and type strains**

| **Isolates** | **ANI(Maximum)** | **Type strain** | **Genebank ID of type strain** |
| --- | --- | --- | --- |
| TK-F8A | 99.99 | *L. reuteri* DSM 20016 | GCA_000016825.1 |
| TK-F8B | 97.54 | *L. rhamnosus* DSM 20021 | GCA_001435405.1 |
| TK-J7A | 97.62 | *L. helveticus* DSM 20075 | GCA_000160855.1 |
| TK-P2A | 99.06 | *L. plantarum* subsp. plantarum ATCC 14917 | GCA_000143745.1 |
| TK-P3A | 99.40 | *S. thermophilus* ATCC 19258 | GCA_004354505.1 |
| P3MRA | 97.36 | *L. delbrueckii* subsp. delbrueckii DSM 20074 | GCA_001433875.1 |
| TK-P4A | 98.31 | *L. paracasei* subsp. paracasei ATCC 25302 | GCA_000159495.1 |
| TK-P4B | 98.89 | *E. faecalis* ATCC 19433 | GCA_000392875.1 |
| TK-P5D | 94.86 | *E. faecium* NCTC7171 | GCA_900447735.1 |
| TK-J6A | 99.99 | *B. animalis* subsp. lactis DSM 10140 | GCA_000022965.1 |

**Table S6.** Strain typing by SNP distance (<100bp)

| **isolates** | **SNP distance（Min）** | **Reference genome** |
| --- | --- | --- |
| TK-F8A | 56 | *Lactobacillus reuteri* DSM 20016 |
| TK-F8B | 88 | *Lactobacillus rhamnosus* DSM 14870 |
| TK-J6A | 17 | *Bifidobacterium animalis* subsp. lactis B420 |
| TK-P2A | 78 | *Lactobacillus plantarum* DOMLa |
| TK-P5D | 40 | *Enterococcus faecium* JE1 |

**Table S7.** Strain typing by MLST based on whole genome

| **Isolates** | **Species** | **Seqeuence type(ST)** | **Locus_Allele** |
| --- | --- | --- | --- |
| TK-P4B | *E. faecalis* | ST745 | aroE_24, gdh_89, gki_25, gyd_6, pstS_7, xpt_2, yqiL_17 |
| TK-P5D | *E.faecium* | ST812 | adk_27, atpA_6, ddl_6, gdh_4, gyd_3, pstS_3, purK_4 |
| TK-P3A | *S.thermophilus* | new | carB_2, clpX_9, dnaA_8, murC_2, pepX_12, pyrG_10, recA_4, rpoB_3 |

**Table S8.** Antibiotic resistance of isolates from probiotic products

| **Isolates** | **Drug Class** | **Annotated antibiotic genes** | **Annotated AMR genes in CARD** | **Query genes in genome** | **AMR Gene Family** |
| --- | --- | --- | --- | --- | --- |
| *B. animalis* ssp. TK-J6A | tetracycline antibiotic | tetW | ARO:3000194 | GFB60_04935 | tetracycline-resistant ribosomal protection protein |
|  | rifamycin antibiotic | Bifidobacterium adolescentis rpoB mutants conferring resistance to rifampicin | ARO:3004480 | GFB60_06860 | rifamycin-resistant beta-subunit of RNA polymerase (rpoB) |
| *E. faecalis* TK-P4B | acridine dye | emeA | ARO:3003551 | GFB65_04525 | multidrug and toxic compound extrusion (MATE) transporter |
|  | diaminopyrimidine antibiotic | dfrE | ARO:3002875 | GFB65_06740 | trimethoprim resistant dihydrofolate reductase dfr |
|  | lincosamide antibiotic;macrolide antibiotic;oxazolidinone antibiotic;phenicol antibiotic;pleuromutilin antibiotic;streptogramin antibiotic;tetracycline antibiotic | lsaA | ARO:3000300 | GFB65_10880 | ABC-F ATP-binding cassette ribosomal protection protein |
| *E. faecium* TK-P5D | aminoglycoside antibiotic | AAC(6')-Ii | ARO:3002556 | GFB66_10865 | AAC(6') |
|  | lincosamide antibiotic;macrolide antibiotic;oxazolidinone antibiotic;phenicol antibiotic;pleuromutilin antibiotic;streptogramin antibiotic;tetracycline antibiotic | msrC | ARO:3002819 | GFB66_12760 | ABC-F ATP-binding cassette ribosomal protection protein |

**Table S9.** Annotated virulence factors in genomes of probiotic isolates

| **Isolates** | **Virulence factors** | **Virulence factors name** | **Related genes** | **location** |
| --- | --- | --- | --- | --- |
| Streptococcus thermophilus TK-P3A | VFG005365(gi:22537432) | (ssp-5)agglutinin receptor | GFB63_05410 | Chromosome |
| 21 | VFG006078(gi:55821451) | (rgpE)polysaccharide biosynthesis protein/putative glycosyltransferase | GFB63_01490 | Chromosome |
|  | VFG006086(gi:55821454) | (rgpB)polysaccharide biosynthesis protein/putative rhamnosyl transferase | GFB63_01505 | Chromosome |
|  | VFG006081(gi:55823390) | (rmlD)dTDP-4-keto-L-rhamnose reductase | GFB63_01540 | Chromosome |
|  | VFG006082(gi:55821452) | (rgpD)polysaccharide ABC exporter ATP-binding protein | GFB63_01495 | Chromosome |
|  | VFG006084(gi:55821453) | (rgpC)polysaccharide ABC exporter membrane-spanning protein | GFB63_01500 | Chromosome |
|  | VFG006088(gi:55821455) | (rgpA)polysaccharide biosynthesis protein/putative rhamnosyl transferase | GFB63_01510 | Chromosome |
|  | VFG006053(gi:55821123) | (epsB)exopolysaccharide biosynthesis protein | GFB63_09585 | Chromosome |
|  | VFG006047(gi:55821122) | (epsC)exopolysaccharide exporter accessory protein | GFB63_09590 | Chromosome |
|  | VFG006074(gi:55821450) | (rgpF)polysaccharide biosynthesis protein | GFB63_01485 | Chromosome |
|  | VFG006011(gi:116627847) | (STER_1071)Transcriptional regulator | GFB63_09580 | Chromosome |
|  | VFG005999(gi:55821117) | (eps2)exopolysaccharide biosynthesis protein, glycosyltransferase | GFB63_09615 | Chromosome |
|  | VFG005865(gi:24378821) | (SMU.322c)glucose-1-phosphate uridylyltransferase | GFB63_03340 | Chromosome |
|  | VFG005987(gi:55821116) | (eps3)exopolysaccharide biosynthesis protein, glycosyltransferase | GFB63_09620 | Chromosome |
|  | VFG006091(gi:55821457) | (rgpX3)polysaccharide biosynthesis protein, putative transporter | GFB63_01525 | Chromosome |
|  | VFG006031(gi:55821120) | (epsE)exopolysaccharide biosynthesis protein, glycosyl-1-phosphate transferase | GFB63_09600 | Chromosome |
|  | VFG005323(gi:55820142) | (cbpD)cell wall protein precursor, similar to choline binding protein | GFB63_04670 | Chromosome |
|  | VFG005190(gi:55820986) | (fbp54)fibronectin-binding protein-like protein A | GFB63_10100 | Chromosome |
|  | VFG005382(gi:116628017) | (srtA)Sortase (surface protein transpeptidase | GFB63_00540 | Chromosome |
|  | VFG019077(gi:169832817) | (plr/gapA)glyceraldehyde-3-phosphate dehydrogenase, type I | GFB63_03095 | Chromosome |
|  | VFG005556(gi:116627090) | (tig/ropA)FKBP-type peptidyl-prolyl cis-trans isomerase (trigger factor) | GFB63_05295 | Chromosome |
| Enterococcus faecalis TK-P4B | VFG002183(gb\|NP_816134) | (cpsH) lipoprotein | GFB65_09750 | Chromosome |
| 19 | VFG002184(gb\|NP_816135) | (cpsG) MurB family protein | GFB65_09755 | Chromosome |
|  | VFG002181(gb\|NP_816132) | (cpsJ) ABC transporter, ATP-binding protein | GFB65_09740 | Chromosome |
|  | VFG002182(gb\|NP_816133) | (cpsI) UDP-galactopyranose mutase | GFB65_09745 | Chromosome |
|  | VFG002188(gb\|NP_816139) | (cpsC) teichoic acid biosynthesis protein, putative | GFB65_09775 | Chromosome |
|  | VFG002180(gb\|NP_816131) | (cpsK) ABC transporter, permease protein | GFB65_09735 | Chromosome |
|  | VFG002186(gb\|NP_816137) | (cpsE) glycosyl transferase, group 2 family protein | GFB65_09765 | Chromosome |
|  | VFG002187(gb\|NP_816138) | (cpsD) glycosyl transferase, group 2 family protein | GFB65_09770 | Chromosome |
|  | VFG045649(gi:397699480) | (ebpC) Endocarditis and Biofilm-Associated Pilus subunitC | GFB65_04590 | Chromosome |
|  | VFG042977(gb\|NP_814822) | (ebpB) endocarditis and biofilm-associated pilus minor subunit EbpB | GFB65_04585 | Chromosome |
|  | VFG045656(gi:384512836) | (OG1RF_10872) sortase | GFB65_04595 | Chromosome |
|  | VFG045638(gi:384512833) | (OG1RF_10869) von Willebrand factor type A domain-containing protein | GFB65_04580 | Chromosome |
|  | VFG043508(gb\|NP_813892) | (fss1) Enterococcus faecalis surface protein Fss1, fibrinogen binding protein | GFB65_00415 | Chromosome |
|  | VFG045677(gi:384513492) | (fsrB) FsrB protein | GFB65_07965 | Chromosome |
|  | VFG045679(gi:384513493) | (fsrA) FsrA response regulator | GFB65_07970 | Chromosome |
|  | VFG045674(gi:397700123) | (fsrC) sensor histidine kinase FsrC | GFB65_07960 | Chromosome |
|  | VFG002174(gb\|NP_815516) | (gelE) coccolysin | GFB65_07955 | Chromosome |
|  | VFG045665(gi:384512514) | (OG1RF_10550) family 8 polysaccharide lyase | GFB65_03120 | Chromosome |
|  | VFG045663(gi:384513489) | (sprE) SprE protein | GFB65_07950 | Chromosome |
| Enterococcus faecium TK-P5D | VFG045671(gi:529233057) | (EFAU085_00344) LacI family sugar-binding transcriptional regulator | GFB66_01940 | Chromosome |
| 8 | VFG045683(gi:529234457) | (EFAU085_01747) phosphatidate cytidylyltransferase | GFB66_05455 | Chromosome |
|  | VFG042986(gi:209491039) | (pilE) cell wall-associated LPXTG-like protein | GFB66_13435 | Plasmid(CP045603) |
|  | VFG042984(gi:209491034) | (pilF) minor pilin subunit | GFB66_13445 | Plasmid(CP045603) |
|  | VFG042989(gi:209491038) | (ACI49672) putative housekeeping sortase | GFB66_13420 | Plasmid(CP045603) |
|  | VFG042988(gi:209491037) | (pilA) PilA | GFB66_13425 | Plasmid(CP045603) |
|  | VFG042987(gi:209491036) | (ACI49670) putative pilus-dedicated sortase | GFB66_13430 | Plasmid(CP045603) |
|  | VFG042990(gi:209491029) | (ACI49664) putative pilus-dedicated sortase | GFB66_06910 | Chromosome |

**Table S10.** Annotated toxic metabolites in probiotic isolates

| **Isolates** | **Toxic metabolites classification** | **Toxic metabolites associated key enzymes** | **Subject ID(UniProt)** | **Annotated genes** | **Locus** |
| --- | --- | --- | --- | --- | --- |
| *E. faecium* TK-P5D | biogenic amines | tyrosine decarboxylase | A0A4U0ZEQ1 | GFB66_01545 | Chromosome |
|  |  | tyrosine decarboxylase | A0A0M1X7C3 | GFB66_01905 | Chromosome |
|  |  | lactate dehydrogenase | A0A4U9QNC0 | GFB66_07935 | Chromosome |
| *E. faecalis* TK-P4B | biogenic amines | tyrosine decarboxylase | X5NXB3 | GFB65_02130 | Chromosome |
| *L. delbrueckii* P3MRA | D-lactate | D-lactate dehydrogenase | P26297 | GFB67_00380 | Chromosome |
| *L. plantarum* TK-P2A | nitrocompounds | FMN-dependent NADH-azoreductase 1 | Q890E7 | GFB62_00420 | Chromosome |
|  | nitrocompounds | FMN-dependent NADH-azoreductase 2 | Q88Y41 | GFB62_04080 | Chromosome |
|  | D-lactate | D-lactate dehydrogenase | Q88VJ2 | GFB62_08865 | Chromosome |
| *L.* helveticus TK-J7A | D-lactate | D-lactate dehydrogenase | P30901 | GFB61_00340 | Chromosome |
| *L.* reuteri TK-F8A | D-lactate | D-lactate dehydrogenase | P26297 | GFB68_09175 | Chromosome |

**Table S11.** Insertion sequences in the genomes of all isolates

| **Isolates** | **IS family** | **IS ID** | **Location** |
| --- | --- | --- | --- |
| *L. reuteri* TK-F8A | ISLre2 | ISLre2 | chromosome |
| *L. rhamnosus* TK-F8B | IS5 | ISLrh2,ISLca2 | chromosome |
| *B. animalis* TK-J6A | ISL3 | IS2001 | chromosome |
|  | IS5 | ISBian1 | chromosome |
| *L. helveticus* TK-J7A | IS110 | ISLhe4 | chromosome |
|  | IS1182 | ISLhe63 | chromosome |
|  | IS200/IS605 | ISLhe65 | chromosome |
|  | IS256 | IS1201 | chromosome |
|  | IS3 | ISLhe6,ISLhe61 | chromosome |
|  | IS30 | ISLdl3,ISLjo1 | chromosome |
|  |  | ISLhe30 | plasmid |
|  | IS607 | ISLhe60,ISLhe9 | chromosome |
|  | IS982 | ISLh1,ISLhe5,ISLhe7 | chromosome |
|  | ISL3 | IS1165,ISLhe2 | chromosome |
|  |  | IS1165 | plasmid |
|  | ISLre2 | ISLde1,ISLhe10,ISLhe11,ISLhe13,ISLke1 | chromosome |
| *L. plantarum* TK-P2A | IS1182 | ISP2 | chromosome |
| *S. thermophilus* TK-P3A | IS200/IS605 | ISStrs1 | chromosome |
|  | IS256 | IS1191 | chromosome |
|  | IS3 | ISSth1b | chromosome |
|  | IS5 | IS981,ISSth7 | chromosome |
|  | IS6 | IS1216E | chromosome |
|  | ISL3 | ISSsu6，ISSmu2 | chromosome |
| *L. delbrueckii* P3MRA | IS110 | ISL4,ISLdl4 | chromosome |
|  | IS256 | ISLdl2 | chromosome |
|  | IS30 | ISL7,ISLjo1 | chromosome |
|  | IS4 | ISL5 | chromosome |
|  | ISL3 | ISL3 | chromosome |
| *E. faecalis* TK-P4B | IS3 | IS1485 | chromosome |
|  | IS256 | ISLgar5 | chromosome |
|  | IS6 | IS1216E | chromosome |
| *E. faecium TK-P5D* | IS256 | IS1542 | chromosome |
|  |  | IS1542,ISLgar5,ISEfa13 | plasmid |
|  | IS3 | IS1485 | chromosome |
|  |  | IS1485 | plasmid |
|  | IS6 | IS1216E | plasmid |
|  | IS982 | ISEfm1 | chromosome |
|  |  | ISEfm1 | plasmid |

**Table S12.** Transposon in the genomes of isolates from probiotic products

| **Isolates** | **Location** | **Query_start** | **Query_end** | **Subject_id** | **Identity** | **Query coverage** |
| --- | --- | --- | --- | --- | --- | --- |
| *E. faecalis* TK-P4B | Chromosome | 2193052 | 2194656 | Tn5385 | 98.009 | 100% |

**Table S13.** Active prophages in the genomes of isolates from probiotic products

| **Isolates** | **Candidate ID** | **Start** | **End** | **Length** | **Category** | **Score** | **Closest phage** |
| --- | --- | --- | --- | --- | --- | --- | --- |
| *L. paracasei* TK-P4A | P4A_10 | 826736 | 872822 | 46087 | Active | 0.81 | Lactobacillus phage PLE2 |
|  | P4A_13 | 960445 | 986303 | 25859 | Active | 0.99 | Lactobacillus phage PLE2 |
|  | P4A_18 | 1153759 | 1192339 | 38581 | Active | 0.98 | Lactobacillus phage PLE3 |
|  | P4A_51 | 2962685 | 2986620 | 23936 | Active | 0.88 | Enterococcus phage IME-EFm5 |
| *L. reuteri* TK-F8A | F8A_23 | 872484 | 893283 | 20800 | Active | 0.95 | Lactobacillus phage Lv-1 |
|  | F8A_24 | 877069 | 931688 | 54620 | Active | 0.95 | Lactobacillus phage phiadh |
|  | F8A_33 | 1185760 | 1228817 | 43058 | Active | 0.9 | Staphylococcus phage IME1323_01 |
|  | F8A_34 | 1216275 | 1246846 | 30572 | Active | 0.98 | Lactobacillus phage LF1 |
|  | F8A_41 | 1429982 | 1440141 | 10160 | Active | 0.85 | Staphylococcus prophage phiPV83 |
| *E. faecalis* TK-P4B | P4B_43 | 2143218 | 2173827 | 30610 | Active | 0.85 | Staphylococcus phage 2638A |
| *E. faecium* TK-P5D | P5D_21 | 1028482 | 1055487 | 27006 | Active | 0.82 | Enterococcus phage phiEf11 |
|  | P5D_42 | 1977586 | 2017670 | 40085 | Active | 0.99 | Enterococcus phage phiFL1B |
|  | P5D_59 | 13025 | 41024 | 28000 | Active | 0.99 | Streptococcus phage phiJH1301-2 |

**Table S14.** Plasmids in the probiotic isolates

| **Isolates** | **Number of Plasmids** | **Virulence factors in Plasmids** |
| --- | --- | --- |
| *L. plantarum* TK-P2A | 2 | -- |
| *S. thermophilus* TK-P3A | 1 | -- |
| *L. delbrueckii* P3MRA | 0 | -- |
| *L. paracasei* TK-P4A | 3 | -- |
| *E. faecalis* TK-P4B | 3 | -- |
| *E. faecium* TK-P5D | 1 | GFB66_13435,GFB66_13445,GFB66_13420,GFB66_13425,GFB66_13430 |
| *B. animalis* subsp. lactis TK-J6A | 0 | -- |
| *L. helveticus* TK-J7A | 2 | -- |
| *L. reuteri* TK-F8A | 0 | -- |
| *L. rhamnosus* TK-F8B | 2 | -- |

**Table S15.** Crispr-Cas systems in the genomes of isolates from probiotic products

| **Isolates** | **Arrays** | **ID** | **Start position** | **End position** | **Length** | **Repeat consensus/ Cas type** | **Repeat length or Cas system length** | **Spacers number** | **Evidence_Level** |
| --- | --- | --- | --- | --- | --- | --- | --- | --- | --- |
| *L. rhamnosus* TK-F8B | Crispr | F8B_C_1 | 1121849 | 1121992 | 143 | AGGTCCTTACACGCAGACTTCTGCGCCGGGGAGCGCGTTATGG | 43 | 1 | 1 |
|  |  | F8B_C_2 | 1306930 | 1307044 | 114 | CTTACATTCTAGCTTACGCCCATAACGC | 28 | 1 | 1 |
|  |  | F8B_C_3 | 1572074 | 1572195 | 121 | GATGCTGCTGCGAAAACGCGCTTACAGTCGCAGCAATCTCC | 41 | 1 | 1 |
|  |  | F8B_C_4 | 1868959 | 1869104 | 145 | GGCCTTAAACGTGATGGCCCGGGTTTGGTCATTGCGTTTAAGGT | 44 | 1 | 1 |
|  |  | F8B_C_5 | 2260989 | 2263270 | 2281 | GTTCTTGAACTGATTGATCTGACATCTACCTGAGAC | 36 | 34 | 4 |
|  | CAS system | F8B_Cas_1 | 2263291 | 2269433 | 6143 | CAS-TypeIIA | \ | \ | \ |
| *B. animalis* subsp. lactis TK-J6A | Crispr | J6A_C_1 | 183330 | 183715 | 385 | GGTCGGTTTTGCGGATGCTTCCG | 23 | 7 | 2 |
|  |  | J6A_C_2 | 219107 | 219190 | 83 | TTTCCGTCTCTTTTTACGCAGAA | 23 | 1 | 1 |
|  |  | J6A_C_3 | 1516909 | 1518527 | 1618 | CCCTCAATGAAGCTCCGAAGCCGAGACTTCGGAGAT | 36 | 22 | 4 |
|  |  | J6A_C_4 | 1917287 | 1917377 | 90 | TTGGCGTCCATGTCACGCGCGGAATCGAT | 29 | 1 | 1 |
|  | CAS system | J6A_cas_1 | 1508634 | 1516856 | 8223 | CAS-TypeIU | \ | \ | \ |
| *L. helveticus* TK-J7A | Crispr | J7A_C_1 | 581211 | 583442 | 2231 | GTCGCACTCCTTGTGAGTGCGTGGATTGAAAT | 32 | 33 | 4 |
|  |  | J7A_C_2 | 1954852 | 1955004 | 152 | TTTGAACAAAACACATCATATACCATATTAGTTCGAATATCAAACTATT | 49 | 1 | 1 |
|  |  | J7A_C_3 | 6197 | 6352 | 155 | AAACCCTTGCTACGAGCGAAAGTCTAAAAAATAGAC | 36 | 1 | 1 |
|  | CAS system | J7A_cas_1 | 572943 | 581049 | 8107 | CAS-TypeIC | \ | \ | \ |
| *L. plantarum* TK-P2A | Crispr | P2A_C_1 | 2666083 | 2666168 | 85 | TAAGAAACTTAAAGTGTCTTATT | 23 | 1 | 1 |
| *S. thermophilus* TK-P3A | Crispr | P3A_C_1 | 710261 | 710342 | 81 | AATAACATTCAAGTGTTTGTTTGAATA | 27 | 1 | 1 |
|  |  | P3A_C_2 | 1555548 | 1557827 | 2279 | GTTTTTGTACTCTCAAGATTTAAGTAACTGTACAAC | 36 | 34 | 4 |
|  | CAS system | P3A_cas_1 | 1550756 | 1554436 | 844176 | CAS-TypeIIC | \ | \ | \ |
| *L. delbrueckii* P3MRA | Crispr | P3MRA_C_1 | 40816 | 40972 | 156 | ATACTGAAAAATACAGAAAATGAAACTATTAAACAATTAGCAGCTGAAAAGCTAC | 55 | 1 | 1 |
|  |  | P3MRA_C_2 | 266065 | 266203 | 138 | AGGCCAGCTCTAAGTCGGCGGCTAAATCCGCGGCCAGTTCTGCCGTTAGTTC | 52 | 1 | 1 |
|  |  | P3MRA_C_3 | 687159 | 687285 | 126 | CTGAGGCTAGAGCCGGTGAAGTTGACCCGGAGGCCGTT | 38 | 1 | 1 |
|  |  | P3MRA_C_4 | 754631 | 757709 | 3078 | GTATTCCCCACGCAAGTGGGGGTGATCC | 28 | 50 | 4 |
|  |  | P3MRA_C_5 | 1771352 | 1771454 | 102 | GTTGGCGTAGGGGAGTGAGAACGATGAAGATCGAT | 35 | 1 | 1 |
|  | CAS system | P3MRA_cas_1 | 745248 | 754601 | 9354 | CAS-TypeIE | \ | \ | \ |
| *L. paracasei* TK-P4A | Crispr | P4A_C_1 | 660742 | 660874 | 132 | GTTTGCGCGTTTCGGAGTTCAGCAATATCAGGCAGGGCTTTGGTCGTTT | 49 | 1 | 1 |
|  |  | P4A_C_2 | 2269004 | 2269826 | 822 | GCTCTTGAACTGATTGATTCGACATCTACCTGAGAC | 36 | 12 | 4 |
|  | CAS system | P4A_cas_1 | 2269851 | 2276010 | 6160 | CAS-TypeIIA | \ | \ | \ |
| *E. faecalis* TK-P4B | Crispr | P4B_C_1 | 1695170 | 1695337 | 167 | GTTTTGGTACCATTCTAAACAACATGACTCTAAAAC | 36 | 2 | 1 |
|  |  | P4B_C_2 | 1878085 | 1878198 | 113 | ACAACGTTCCCTTTGGTCACCTTGTGCTGTTC | 32 | 1 | 1 |
| *E. faecium* TK-P5D | Crispr | P5D_C_1 | 1685269 | 1685362 | 93 | TGAGGAAGAAGGTGTTGTTTCTGCTGCA | 28 | 1 | 1 |
|  |  | P5D_C_2 | 2341987 | 2342099 | 112 | CTTTGCTCAACCAGTATTGTTTATCATCT | 29 | 1 | 1 |
|  |  | P5D_C_3 | 430 | 557 | 127 | TCAGAGGGTATGAAAATCATACC | 23 | 2 | 1 |
